# Lymphatic leaks – success of intranodal lymphangiogram first strategy

**DOI:** 10.1186/s42155-024-00499-7

**Published:** 2025-01-09

**Authors:** Alan Campbell, Diana Velazquez-Pimentel, Matthew Seager, Richard Hesketh, Julian Hague, Jowad Raja, Jocelyn Brookes, An Ngo, Miles Walkden, Anthie Papadopoulou, Daron Smith, Borzoueh Mohammadi, Ravi Barod, Mohammed Rashid Akhtar, Jimmy Kyaw Tun, Deborah Elise Low, Ian Daniel Renfrew, Tim Fotheringham, Conrad von Stempel

**Affiliations:** 1 University College London Hospital NHS Foundation Trust, London, UK; 2 Barts Health NHS Trust, London, UK; 3 King’s College Hospital NHS Foundation Trust, London, UK; 4 Royal Free London NHS Foundation Trust, London, UK; 5 Division of Surgery and Interventional Science, University College London, London, UK; 6 National University Hospital, Singapore, Singapore

**Keywords:** Lymphangiogram, Lymphatic, Chyle

## Abstract

**Background:**

Lymphatic leaks are associated with significant mortality and morbidity. Intranodal lymphangiography (ILAG) involves the direct injection of ethiodised lipid into the hilum of lymph nodes. It is diagnostic procedure that can have therapeutic effects secondary to a local sclerosant effect. The aim of the study is to describe the technical and clinical success of ILAG and adjunctive lymphatic interventions performed as first line interventional techniques for lymphatic leaks refractory to conservative and medical management in a multicentre cohort of patients with symptomatic large volume lymphatic leaks.

**Methods:**

Multicentre retrospective study of all lymphatic interventions performed between 2017–2023 in patients with large volume lymphatic leaks (> 500 ml a day). Intranodal lymphangiography was performed initially with technical success defined as opacification of the lymphatics at the aortic bifurcation and demonstration of lymphatic leak on the index ILAG procedure or immediate post procedural CT was recorded. Lymphatic embolisation was performed with a combination of direct puncture or transvenous cannulation with glue and or coil embolisation of the thoracic duct or leak point and in cases with refractory leak. Clinical success was defined as reduction in drain output to less than 20 mL per 24 h, or no further insensible lymph leak. Time to clinical success after ILAG and adjunctive embolisation was recorded.

**Results:**

ILAG alone lead to clinical success in 14 of 32 (44%) patients after a median of 14 days. Subsequent embolisation was performed in 12 refractory cases; this was successful in 8 (67%) at median of 8 days. Overall clinical success of all lymphatic interventions was 69% (22 of 32 patients) at a median of 11 days (IQR 5–34). No statistically significant correlation between the site of leakage, aetiology or embolisation technique correlated with clinical success. Decision to proceed to repeat ILAG or an adjunct procedure was made on a clinical basis, following multidisciplinary discussion.

**Conclusions:**

ILAG can be employed a first line interventional therapeutic technique to treat clinically significant lymphatic leaks that are refractory to conservative and medical management. Adjunctive procedures, including embolisation, can be considered as part of clinical decision making after a period of 1–2 weeks’ watchful waiting in continuingly refractory cases.

## Background

Lymphatic leaks complicate up to 10% of surgery, most commonly after thoracic, oesophagogastric and retroperitoneal surgery and can result from trauma, infections and malignant lymphatic invasion. Clinically large volume leaks may manifest as chylothorax, chylous ascites, chyluria, or a lymphocoele. The loss of lipid-rich lymphatic fluid, results in protein, fat-soluble vitamin loss and electrolyte losses, causing significant associated morbidity and mortality, including principally nutritional-associated immunosuppression and disrupted surgical wound-healing [1].

Moderate to low volume leaks, of less than < 500 mL per day, are often treated with dietary fat restriction [2] fat-soluble vitamin supplementation and octreotide treatment, which reduces thoracic duct flow and inhibits pancreatic exocrine function [3]. High output leaks (> 500 ml per day) may require surgical or interventional procedures. Identification of the level of lymph leak is performed with radionuclide lymphoscintigraphy, MRI lymphangiography and lymphangiography from a pedal lymphatic cut down and cannulation [4]. Intranodal lymphangiography (ILAG) is a recently developed technique where the hilum of, most commonly, inguinal lymph nodes are cannulated under ultrasound guidance and lipiodol (Lipiodol® ultrafluid a 48% iodinated ethyl ester of poppy seed oil; Guerbet Laboratoires, France) is slowly injected with monitoring of progression by fluoroscopy [5, 6]. Lipiodol lymphangiography is used as a diagnostic agent to identify leaks and may have a therapeutic effect by causing localised fat saponification at the site of leak (i.e. local sclerosant effect) [7, 8]. Adjunctive techniques may be required with surgical or interventional radiological procedures including direct lymphatic ligation and fluoroscopic guided lymphatic sclerosis, embolisation and ligation. [1, 9]. A combination of lymphangiography, direct puncture and embolisation of the leak point and/or thoracic duct has been shown to effectively treat chyle leaks arising at different locations [6, 10–12].

The aim of the study is to describe the technical success and clinical success of ILAG and adjunctive lymphatic interventions performed as first line interventional techniques for lymphatic leaks refractory to conservative and medical management in a multicentre cohort of patients with symptomatic large volume lymphatic leaks.

## Methods

Retrospective study of all patients treated with ILAG and adjunctive lymphatic interventions between January 2017 to December 2023 across four large teaching hospitals that in combination are the tertiary referral centres for head and neck, trauma, cardiothoracic, oesophageal, vascular, gynae-oncology, uro-oncology surgery and tropical diseases. Data was collected from electronic health records and PACS.

### Inclusion criteria

Cases included in this retrospective observational study were of iatrogenic or idiopathic chyle leaks (from any anatomical site), with clinical and/or biochemical confirmation of chylous content of the leak and which were refractory to conservative management strategies.

### Exclusion criteria

Cases wherein ILAG was performed to diagnose suspected lymphatic malformations and lymphangectasia were excluded.

### ILAG technique

ILAG was performed after obtaining informed written consent. Local anaesthetic (1% Lidocaine 5 mL) was instilled into the subcutaneous tissues over each groin overlying the target lymph nodes. Ultrasound guided cannulation of the inguinal lymph node hila was performed with a 24-gauge (24G) spinal needle, or similar. Test injections with 2–3 mL of iodinated contrast (Omnipaque®, Iohexol – GE Healthcare, USA) was performed to confirm cannulation of draining lymphatics as opposed to inadvertent efferent vein. Up to 30 mL lipiodol was instilled to a single node on each side over approximately 30 min by hand injection (approximately 0.2 to 0.7 mL/min). Intermittent fluoroscopy was used to monitor progression of lipiodol into the pelvis, abdomen and chest. An unenhanced CT chest, abdominal and pelvis scan was performed at a 1-h post-procedure interval to assess for any lipiodol extravasation at the site of suspected leak.

Repeat ILAG was considered in select cases following multidisciplinary discussion where a reduction in leak was observed after initial attempt but not clinically felt enough to require empirical embolisation.

### Adjunctive procedures (ILAG +)

Embolisation or direct sclerosis of lymphatics via either direct nodal, cisterna chyli or thoracic duct puncture was performed in cases with refractory leaks following multidisciplinary discussion. After ILAG, patients were observed for a up to 14 days before attempting embolisation. A repeat ILAG was performed and needle guidance performed with a combination of cone beam CT or standard CT guidance and fluoroscopy using a Chiba 20G needle. In selected cases, thoracic duct cannulation and embolisation was performed from either a transvenous or percutaneous transabdominal puncture of the cisterna chyli and cannulation with a 0.018 inch guidewire (V18™ – Boston Scientific, USA) and 2.6 Fr catheter (CXI® – Cook, USA). Embolisation was performed with glue (Glubran – GEM, Italy; Magic Glue – Balt Group, France or Histoacryl – B. Braun Medical, Sweden) and lipiodol (in a 1 to 4 ratio) and with or without a platinum coil scaffold (Ruby – Penumbra, USA).

### Endpoints

Patients were followed up by referring clinical teams with a combination of patient reported symptoms and imaging with CT, radiography and/or ultrasound for at least 3 months after the index admission and until censorship date or death. Technical success of ILAG was defined as opacification of the paraortic lymphatics at the level of the aortic bifurcation. Demonstration of lymphatic leak on either the index ILAG procedure or immediate post procedural CT was recorded as a secondary technical end point. Clinical success was defined as reduction in drain output to less than 20 mL per 24 h [13] or when there was a resolution of visible chyluria or other insensible lymph leak. The CIRSE Classification System for Complications was used for the grading of documented complications [14].

### Statistical methods

Data was analysed using descriptive statistics and non-parametric univariate analyses.

### Ethical consideration

This study was a retrospective audit of standard clinical practice and did not require formal ethical committee registration.

## Results

Thirty-two patients were included: 18 patients were male (56%) and 14 (44%) were female; median age was 68 (range 28 to 79 years, quartile 1: 12.75, quartile 3: 70.75).

In all cases, patients were referred for ILAG following the failure of conservative and medical measures for at least 14 days and after multidisciplinary team discussion. Patients were considered for lymphatic intervention if the leak volume exceeded 500 mL per day and/or where the patients were highly symptomatic due to the lymphatic leak (including lymphatic loss manifesting as weight loss and/or immune compromise).

### Aetiology

The underlying aetiology and site of lymphatic leaks are described in Table 1. Most cases were of iatrogenic aetiology (n = 24, 75%), followed by idiopathic cases (*n* = 7, 22%) and a post-traumatic case (*n* = 1, 3%).

**Table 1:** Aetiologies of chyle leaks and count of patients

Category	Presentation/leak manifestation	Sub-category	Count
Idiopathic	Chylous ascites	Alcohol-related pancreatitis	1
	Chylothorax	Diffuse large B-cell lymphoma	1
		Mantle cell lymphoma	2
		Non-Hodgkin's lymphoma	1
		Tuberculosis	1
	Chyluria	Idiopathic	1
Iatrogenic	Chylous ascites	Gynaecological exenteration	1
		Nephrectomy	2
		Retroperitoneal mass resection	1
		Retroperitoneal lymph node clearance	1
	Chylothorax	Open aortic valve replacement	1
		Oesophagectomy (open Ivor Lewis)	5
		Pleurectomy (malignant mesothelioma)	2
		Pulmonary lobectomy	2
	Chyluria	Neobladder cystoplasty	1
	Lymphocoele	Femoral dialysis access surgery	1
		Cervical lymph node dissection	3
		Thyroidectomy	1
		Oesophagectomy	1
		Radical prostatectomy	1
		Renal transplant	1
Traumatic	Lymphocoele	Groin shotgun injury	1

### Procedural complications

Following the CIRSE Classification System for Complications [14, 15] there was one case involved a Grade 1a complication, where small volume of intravenous lipiodol intravasation was observed with no clinical sequela. No long-term complication of lymphatic intervention was identified including lower limb lymphoedema.

### Procedural outcomes

**Fig. 1:**
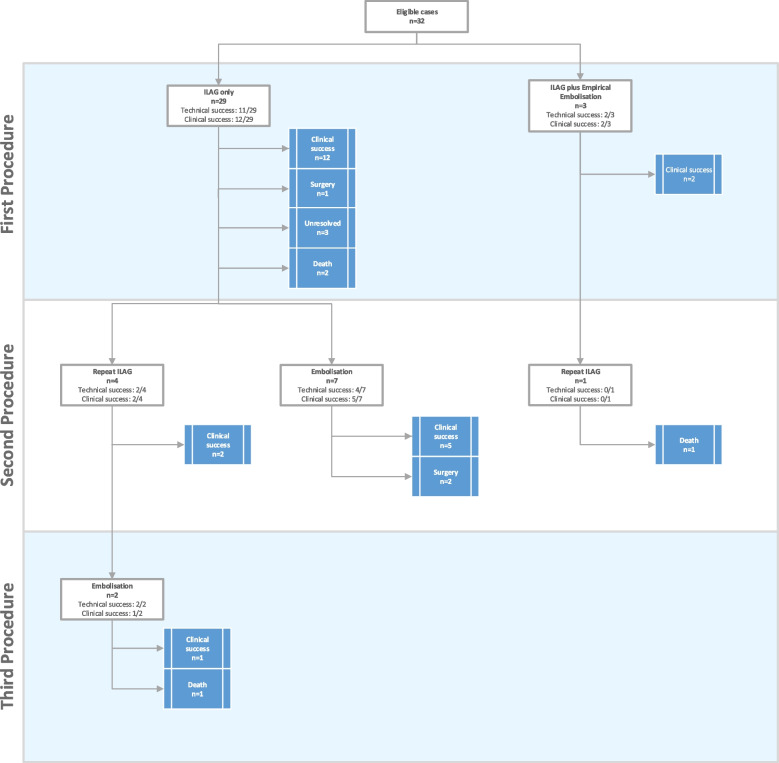
Patient procedural progression, as per the STARD 2015 reporting guidelines [16]

Forty-two ILAG procedures were carried out in 32 patients. Of the cases included, 63% (n = 20/32) patients underwent ILAG only (including 8 who underwent a repeat ILAG only), and 38% (n = 12/32) also underwent at least one adjunctive procedure. Figure 1 demonstrates the patient procedural progression.

A median of 8 mL (range 2–30 mL) of lipiodol was instilled per puncture. ILAG technical success was achieved in 91% of patients (*n* = 29/32), and 93% of ILAG procedures (*n* = 39/42). Technical failure occurred in 3 cases due to no suitable lymphatic access (*n* = 2) and opacification of lymphatic vessels to level of iliac only (*n* = 1). Lymphatic leak was observed during initial ILAG/immediate post ILAG CT scan in 59% of patients (*n* = 19) but not in the other 41% (*n* = 13) cases.

In 12 cases an adjunctive embolisation was attempted after ILAG: 9 embolisation procedures were technically successful; in 3 cases embolisation was not technically feasible due to inability to cannulate the thoracic duct from either a percutaneous transabdominal or transvenous route. Embolisation was carried out at a median of 6 days (quartile 1: 3.75; quartile 3: 8.75) of initial ILAG when there was ongoing significant leakage. Overall, time between repeat/additional procedures varied, from days to several weeks (range of 1 day to 33 weeks). However, most (*n* = 10 s procedures; *n* = 3 third procedures) repeat or further procedures occurred after a period of watchful waiting of up to 2 weeks. Early re-intervention (< 1 week) was considered following multidisciplinary team discussion for unstable patients where lymphatic leak was identified as a major driver for clinical deterioration (*n* = 8 s procedures; *n* = 2 third procedures). No precise robust threshold based on time since intervention alone could be derived from this data. Following repeat intervention a progressive decrease in leak was seen in successful cases.

**Fig. 2:**
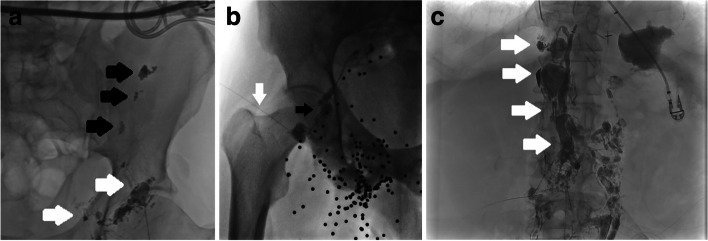
Representative images from three separate cases included in this study. **a** Demonstrates a left ILAG in patient with large lymphocoele post prostatectomy; lipiodol and glue was injected into the adjacent lymph node (white arrows) with beads of glue leaking into the lymphocoele in the left iliac fossa (black arrows); a drain seen within the lymphocoele. **b** This patient had previously had a shotgun injury, evident from the shot pellets projected over the groin in this fluoroscopic image, and a nearby lymph node was cannulated (needle, white arrow) and with visualisation of the lymphocoele (black arrow). **c** Demonstrates a case wherein the cisterna chyli (white arrows) was opacified, punctured via a transhepatic approach and embolised with coils and glue

Median clinical follow up was 44 weeks from the first ILAG procedure (IQR 19.4—94). Four patients died within 90 days of referral from multiorgan failure related to their underlying diagnosis. Clinical success with resolution of the lymphatic leak was seen in 69% (*n* = 22/32) of patients after a median of 15 days (IQR 7–51). ILAG alone led to a resolution of lymphatic leak in 44% of cases (*n* = 14/32): in 38% (*n* = 12/32) after a single ILAG. Eight patients underwent a repeat ILAG and in 6% (*n* = 2/32) this led to resolution of the leak at a median of 11 days. Embolisation led to clinical resolution of the leak in eight cases (*n* = 8/32) after a further median of 9 days. In 6% (*n* = 2/32) of cases surgical ligation/cautery of the leak was required after failure of both ILAG and subsequent embolisation attempts. In one of these cases a methylene blue/lipiodol ILAG was performed in theatre, with subsequent thoracotomy and direct application of glue to the visible lymphatic leak. Comprehensive procedural details are described in Table 2 and example cases in Fig. 2. The overall data and median time to resolution is summarised in Table 3.

**Table 2:** Procedural details

	n
**A) ILAG technique – puncture location (where documented, *n* = 21/32)**	
Unilateral inguinal node	16
Bilateral inguinal nodes	2
Jugular lymph node	1
Retroperitoneal lymph node	1
Axillary lymph node	1
**B) Attempted adjunctive procedure types (*n* = 12/32):**	
Repeat ILAG	4
Direct leak puncture and embolisation	4
Cysterna chyli puncture and antegrade thoracic duct embolisation	3
Transvenous cannulation and retrograde thoracic duct embolisation	5
**C) Other procedures (*n* = 3)**	
Surgical management	3

**Table 3:** Procedural outcomes

	**Total patient cases n**	**Technical success** **n (%)**	**Technical failure** **n (%)**	**Clinical success** **n (%)**	**Clinical failure** **n (%)**	**Time to resolution from last procedure/days**	
						**Median**	**Interquartile range**
**Procedure type**							
ILAG alone (including repeat ILAG)	20	17 (85%)	3 (15%)	14 (70%)	6 (30%)	14	6–34
Embolisation	12	9 (75%)	3 (25%) *	8 (67%)	4 (33%)	8	5–30
Overall	32	29 (91%)	3 (9%)	22 (69%)	10 (31%)	11	5–34

Using Chi-square with Yate’s modification, Fisher’s exact test and non-parametric univariate analyses, there was no statistically significant correlation between the clinical outcome (success or failure of resolution of leak) and the underlying aetiology, site of leak, presence of active leaking on the initial ILAG or embolisation technique (*P* > 0.15).

## Discussion

Lymphatic leakage is uncommon and seen in up to 5% of oesophagectomie [17] 6.2% of head and neck surgery including thyroidectomy with node clearance [2, 18] 7% of colorecta [19] and 9% of gynaecological surgery [20]. The majority of which are small volume and self-resolve. Other aetiologies include filarial infection resulting in chyluria, trauma to lymphatics and malignant lymphatic invasion (primarily non-Hodgkin’s lymphoma) [21–24]. Lymphatic leaks and the associated metabolic losses can reduce wound healing and slow down recovery from surgery and in extreme cases can be fatal. For example, it has been shown that there is fivefold increase in in-hospital and 30-day mortality in patients with post-oesophagectomy chylothora [1] and an overall 40–70% increase in mortality with patients with chylous ascites [17]The natural history of lymphatic leaks is difficult to characterise as many small leaks may never be diagnosed and only large volume leaks (> 500 ml per day) are intervened on. Early intervention, particularly in chylothorax, improves patient outcomes as delayed intervention has high morbidity and late surgical intervention can be technical challenging [25]. Minimally invasive techniques are therefore preferred to manage lymphatic leakage. The proposed mechanism of the therapeutic effect of lymphangiography is at the site of lipiodol extravasation, there is a local inflammatory response leading to local fat saponification and sclerosis leading to sealing the leak within days to weeks [7, 8, 26]. This mechanism would benefit from further investigation in future work.

This retrospective study demonstrates that a stepwise approach to large volume lymphatic leaks with ILAG and subsequent embolisation for refractory cases is effective in 69% of patients with lymphatic leaks. ILAG alone can be both diagnostic and therapeutic, this study showed that ILAG led to cessation of leak in 44% of cases (*n* = 14/32) at a median time of 11 days. Subsequent adjunctive embolisation can further increase clinical success (67%, 8/12 cases) at a median of 9 days from embolisation. This is similar to that described in the literature where clinical success of lymphangiography ranges from 51–88%, the difference may be accounted for by variability in aetiology, anatomical challenges, procedural variables (e.g. rate or volume of lipiodol administered) or early multidisciplinary discussion [7, 26, 27]. The median lipiodol administered in this study was 8 mL (range 2–30 mL); this was likely less than the described in some other published, for example, the 29 mL (range 8–60 mL) mean volume described in a recent study [27].

With the addition of embolisation, 80% clinical success has been reported [28–31]. The time to resolution with ILAG coupled with glue embolisation ranges from 7–10 days [2, 18]. Time to resolution varied throughout the sample for all leak volumes and aetiologies. No precise robust threshold based on time since intervention alone could be derived from the data. Decisions to proceed to repeat ILAG or to adjunctive embolisation procedures were made through multidisciplinary clinical discussion. This included consideration of volume of leak (with high leak rates often prompting earlier action), the presence of an additional lymph node to target for a repeat ILAG procedure, and post ILAG showing a good target for a percutaneous target to access the cysterna chylii or thoracic duct. In this cohort no difference in leak aetiology, site or interventional technique was associated with a statistically significant difference to overall time to resolution.

No precise time-based threshold for considering further intervention is derived from this study. However, the following considerations are suggested based on the experience from these cases:

- Lymphatic leaks can be managed using an ILAG-first approach, although clinicians should be prepared to consider adjunct and alternative procedures;
- Even in procedures which are overall successful, leaks may take a long time to resolve, which should form a routine part of patient counselling.
- Decisions to proceed to repeat, adjunct or alternative procedures should be taken through multidisciplinary discussion (e.g. interventional radiologists, surgeons, physicians and dieticians), individualising management plans by considering the patient’s comorbidities, nutrition status, leak volume and overall clinical progress;
- Where there is clinical deterioration, the decision to reintervene should occur within 1–2 weeks. In the absence of clinical deterioration, premature re-intervention should be avoided if clinically acceptable assume leaks will resolve in weeks to months after the procedure.

Further prospective studies should focus on methodologies that can quantify this as a primary endpoint, i.e. drain output data and indirect quantification of insensible leaks such as chyluria. Furthermore, the heterogenous indications for ILAG procedure presents a clinical challenge, future prospective studies should have broad inclusion criteria at trial sites that have strong interdisciplinary relationships (e.g. thoracic surgery and radiology) to be successful at recruiting patients to a prospective study.

Limitations.

This study is limited by heterogenous aetiologies and incomplete or limited documentation (for example drain output data could not be calculated in patients with insensible lymph leak such as chyluria). Furthermore, the natural history of lymphatic leaks has not been fully evaluated as it was not possible to identify a comparator control group with large volume leaks treated with conservative measures alone. Similarly, given that there are no established clinical guidelines available at the outset of this study, decision to repeat ILAG or proceed with secondary interventions were based on multidisciplinary team discussion alone.

The retrospective nature of this study and the small patient numbers limit the generalisability of the presented findings and increases the risk of false negative results (type II error). Further, no comparative study between intranodal ethiodised oil injection versus intranodal glue injection or redo surgery was performed.

## Conclusions

ILAG has a high technical success rate and as an initial therapeutic technique can lead to closure of even large volume lymphatic leaks refractory to conservative and medical management. Additional lymphatic embolisation further improves clinical success. The authors suggest that in patients with a technically successful ILAG, a strategy of watchful waiting should be adopted for approximately 1–2 weeks, if clinically appropriate, and more invasive procedures, such as embolisation, can be considered for refractory cases.

## Data Availability

The datasets generated and/or analysed during the current study are not publicly available due potential patient identifiability and limited utility of the remaining data beyond that published but are available from the corresponding author on reasonable request.

## Abbreviation

ILAG: Intranodal lymphangiography
